# Reply from “Trinity University,” Toronto

**Published:** 1904-04

**Authors:** Robert J. Reade

**Affiliations:** Secretary Board of Dental Studies, Trinity University


### Domestic Correspondence.

### REPLY FROM "TRINITY UNIVERSITY," TORONTO.

To the Editor:

Sir,—I am happy to be able to assure you that the fears expressed in your editorial entitled "Novel Method in conferring Degrees," which appeared in the February issue of the International Dental Journal, have no foundation whatever in the true facts of the case. As it seems evident from your article that you have been misinformed in several important points, I hope you will allow me to lay the facts before you.

A careful study of the curriculum prescribed by Trinity University, Toronto, for the degree of Doctor of Dental Surgery, and the regulations in connection with the same, all of which I enclose herewith, will, I feel sure, lead you to recognize that the knowledge required to obtain a D.D.S. from this University is equal to that required by the most advanced dental institutions on this continent. Such, at least, was the intention of those who constructed the curriculum, and their purpose was as far removed as possible from the taint of commercialism. In further evidence of this I might mention that the University not only demands proof of thoroughly sound theoretical knowledge and practical ability, but also requires that a candidate, in order to hold a Trinity D.D.S., must pledge himself to observe the ethical side of his professional life. This is shown by the following declaration, which must be subscribed by all candidates before receiving the degree:

"All candidates who successfully pass the Final Examination for the Degree of D.D.S. will be required to subscribe to the following declaration before receiving their Diploma:

"I, ........................., do solemnly promise that so long as I hold the Diploma of the University of Trinity College, or remain on the rolls as a Graduate in Dentistry, I will not resort to any advertising of a kind that may be adjudged by the said University to be unprofessional, nor will I be guilty of any other practices deemed by them unbecoming to my profession or calculated to bring discredit upon the University.

"And I hereby agree that if, in the opinion of the said University, I shall at any time be shown to have violated this undertaking in any way, I will, if the said University shall so decree, surrender my Diploma and Degree and all rights whatsoever that I may be in the enjoyment of as a graduate of the said University, and will consent to my name being struck off the rolls."

As to the special regulation which apparently led to the writing of the editorial, I am convinced that all that is needed to entirely do away with the objection urged against it is that the facts should be clearly and fully understood. The regulation reads as follows:

"All legally qualified practitioners of Dental Surgery of Great Britain or of any portion of the British Empire will be admitted to the Final Examination without passing further matriculation, and without further attendance on lectures."

Now it must be allowed that this special privilege, whether or not it be deemed a wise one, does not make the degree easy to obtain, nor does it call for a low standard of knowledge on the part of the candidate. The qualification of which the candidate must furnish proof in order to be admitted to the Final Examination, is that he has been licensed by the government of his own country for the practice of dental surgery, and that that country be a part of the British Empire. As a matter of fact all parts of the empire, with only one or two minor exceptions, require regular apprenticeship and a college course in order that a candidate may obtain his license to practise. If in the vast extent of the British Empire there are one or two exceptions to this rule, still it must be admitted, even then, that a candidate from such a district will need to devote himself to a very thorough course of private study, or else spend some considerable time in a dental college in attendance on lectures, before being able to pass the examination prescribed by Trinity University. Indeed, so strict has this examination always been, and so high is the estimation in which it is held among the profession, that only one candidate (and he came from Great Britain itself) has as yet ventured to present himself for examination without previous attendance at college lectures.

The editorial says, " One of the singular conditions demanded in conferring this degree is to be found in the fact that the recipient is not to make use of it in the Dominion of Canada, but it is available only in the other portions of the colonial possessions of Great Britain." How this idea could have arisen I am at a loss to understand. It is an entirely mistaken one, and I know of no ground whatever which could properly have given rise to such a report. As a matter of fact, the University confers, as one would naturally expect, more degrees upon residents of our own Dominion than upon those who come here from other parts of the empire and from abroad, and the degree is freely used and held in high esteem in Toronto itself, throughout the Province of Ontario, and in other parts of the Dominion.

If I am not trespassing too much upon your space, I might refer briefly to one or two other points of lesser importance. Your editorial seems to have confused the universities, which have the power to grant D.D.S. degrees, with the Royal College of Dental Surgeons, which is the body authorized to confer the L.D.S. There is, however, no necessary connection between this college and the several universities, and, as a matter of fact, none whatever between it and Trinity University, although it is in this college that nearly all candidates for university examinations in dentistry in Canada receive their teaching. The College maintains a high standard of work, and in order to obtain the L.D.S., which it alone is authorized to confer in this Province, the candidates not only must have attended four full winter sessions at the College, but also must have been articled to a dentist for three and a half years. It will be seen, therefore, that there is a double misapprehension underlying the implied statement of your editorial that the schools of the Dominion of Canada offer an easy pathway to the D.D.S. degree. For, in the first place, such schools or colleges have no power to confer academic degrees, and in the second place the license which they do confer is hedged about by a thorough and prolonged course of training and by examinations of a high standard.

It may perhaps be well that I should take this opportunity of stating that inasmuch as Trinity University has recently entered into a Federation Agreement with the University of Toronto, the curriculum of the former institution, which I am sending to you herewith, will give place, on and after the first day of October next, to that of the University of Toronto. Consequently, the regulation as to exemptions allowed to regularly qualified practitioners of dental surgery within the British Empire will then cease to be operative, except for candidates previously enrolled, there being no similar provision in the regulations of the University of Toronto. Therefore, even if there were anything to object to in such a provision, it would quickly cease to be a matter of practical concern, by reason of the Federation to which I have referred.

ROBERT J. READE, M.A., D.D.S.,
*Secretary Board of Dental Studies, Trinity University.*

